# Cognitive mapping style relates to posterior–anterior hippocampal volume ratio

**DOI:** 10.1002/hipo.23072

**Published:** 2019-02-03

**Authors:** Iva K. Brunec, Jessica Robin, Eva Zita Patai, Jason D. Ozubko, Amir‐Homayoun Javadi, Morgan D. Barense, Hugo J. Spiers, Morris Moscovitch

**Affiliations:** ^1^ Department of Psychology University of Toronto Toronto Ontario Canada; ^2^ Rotman Research Institute Baycrest Health Sciences Toronto Ontario Canada; ^3^ Institute of Behavioural Neuroscience Department of Experimental Psychology University College London London United Kingdom; ^4^ Department of Psychology SUNY Geneseo Geneseo New York; ^5^ School of Psychology University of Kent Kent United Kingdom

**Keywords:** cognitive map, hippocampus, long axis, spatial navigation, volume

## Abstract

As London taxi drivers acquire “the knowledge” and develop a detailed cognitive map of London, their posterior hippocampi (pHPC) gradually increase in volume, reflecting an increasing pHPC/aHPC volume ratio. In the mnemonic domain, greater pHPC/aHPC volume ratios in young adults have been found to relate to better recollection ability, indicating that the balance between pHPC and aHPC volumes might be reflective of cross‐domain individual differences. Here, we examined participants' self‐reported use of cognitive map‐based navigational strategies in relation to their pHPC/aHPC hippocampal volume ratio. We find that greater reported cognitive map use was related to significantly greater posterior, relative to anterior, hippocampal volume in two separate samples of young adults. Further, greater reported cognitive map usage correlated with better performance on a self‐initiated navigation task. Together, these data help to advance our understanding of differences between aHPC and pHPC and the greater role of pHPC in spatial mapping.

1

The hippocampus has long been proposed to support a spatial‐mnemonic “cognitive map” (Epstein, Patai, Julian, & Spiers, 2017; O'Keefe & Dostrovsky, 1971; O'Keefe & Nadel, 1978; Schiller et al., 2015; Bellmund, Gärdenfors, Moser, & Doeller, 2018). Recent research, however, suggests that the relative contributions of the anterior and posterior hippocampal segments in the formation of this map may differ (Poppenk, Evensmoen, Moscovitch, & Nadel, 2013). Striking results come from analyses of licensed London taxi drivers, who learn the complex road layout of London, UK (“the knowledge”) and navigate it daily. They show greater posterior hippocampal gray matter volumes and smaller anterior hippocampal volumes relative to the general population (Maguire et al., 2000) and to London bus drivers, who drive London's streets daily but don't navigate them (Maguire, Woollett, & Spiers, 2006). Longitudinal data collected over the course of acquiring “the knowledge” specifically suggests that only those taxi drivers who qualified showed an increase in posterior hippocampal grey matter (Woollett & Maguire, 2011).

Whereas these volumetric differences have been reported in a highly specialized population of taxi drivers, real‐world and virtual reality spatial learning studies have suggested that nonspecialized individuals vary in the degree to which they employ “cognitive maps” and that these differences relate to hippocampal volume and activity (Bohbot, Lerch, Thorndycraft, Iaria, & Zijdenbos, 2007; Hartley & Harlow, 2012; Iaria, Petrides, Dagher, Pike, & Bohbot, 2003; Marchette, Bakker, & Shelton, 2011; Schinazi, Nardi, Newcombe, Shipley, & Epstein, 2013; Weisberg & Newcombe, 2016, 2018; Weisberg, Schinazi, Newcombe, Shipley, & Epstein, 2014). In particular, Schinazi et al. (2013) found that right pHPC volume was negatively related to pointing errors made on a task requiring remembering the relative position of landmarks in a spatial environment. Other studies have also found relationships between hippocampal volumes and measures of spatial memory and map‐based strategy use (Bohbot et al., 2007; Hao et al., 2016; Hartley & Harlow, 2012; Iaria et al., 2003; Konishi & Bohbot, 2013; Sherrill, Chrastil, Aselcioglu, Hasselmo, & Stern, 2018; Wegman et al., 2014).

Converging evidence has also been reported in the mnemonic domain. Greater pHPC/aHPC volume ratios were found to relate to better memory across diverse paradigms, including source memory judgments for scenes and recollection responses for pairs of words and pictures, suggesting a trade‐off between the contributions of anterior and posterior hippocampal segments (Poppenk & Moscovitch, 2011). Specifically, the right pHPC/aHPC volume ratio showed a stronger relationship with memory than raw aHPC and pHPC volumes alone. These results suggest that individual differences in complex spatial and mnemonic abilities requiring a richly detailed representation may rely on a larger pHPC, which may entail a smaller aHPC. Based on these previous findings, we chose to focus on the ratio of volumes as our target measure of interest. Given that differences in aHPC and pHPC pathology are found in Alzheimer's disease (AD) and healthy aging (Llado et al., 2018; Ta et al., 2012), a better understanding of the relationship between hippocampal long axis structure and navigational ability may also inform our understanding of pathology progression and protective factors.

Here, we examined the volume ratios of posterior relative to anterior hippocampal segments in two studies of younger adults who completed a navigational strategies questionnaire (NSQ) assessing their reliance on cognitive map strategies (NSQ published in Brunec, Bellana et al., 2018; see Appendix A). This questionnaire was designed to quantify the degree to which individuals rely on map‐based strategies and spatial memories when navigating in the real world. Questions include self‐reported strategies when navigating (i.e., “When planning a route, do you picture a map of your route or do you picture scenes of what you will see along the way?”) and ratings of navigational ability (i.e., “Do you find it easy to read and use maps?”). In the present analyses, we sought to determine, in two independent datasets, if individuals who reported greater use of mental maps (higher mapping scores) had larger pHPC/aHPC volume ratios. We predicted that higher pHPC/aHPC volume ratios should relate to greater reliance on map‐based navigational strategies, consistent with predictions based on previous studies and theories of specialization along the hippocampal long‐axis (Poppenk et al., 2013; Strange, Witter, Lein, & Moser, 2014). This prediction is based on evidence of a trade‐off between aHPC and pHPC function, observed in a range of episodic memory tasks (Poppenk & Moscovitch, 2011) and spatial abilities (Maguire et al., 2000), as well as a strong link between pHPC function and spatial behavior (Fanselow & Dong, 2010; Ryan, Lin, Ketcham, & Nadel, 2010).

The first study (i.e., the Toronto dataset) included 33 participants (mean age 24.3 years, *SD* = 4.26; 22 female). Data were collected for four additional participants, who were excluded (one due to excessive difficulty with the task and three due to incomplete data or technical issues). High‐resolution T1‐weighted structural scans were acquired with a 3 T Siemens TIM Trio MRI scanner at the Rotman Research Institute at Baycrest as part of two related neuroimaging experiments (TR = 2000 ms, TE = 2.63 ms, 1 × 1 × 1 mm^3^ resolution). As six participants participated in both experiments, their volume estimates were averaged across both. Both experiments were approved by the ethics committee at the Rotman Research Institute at Baycrest. Functional data from one of the experiments have previously been reported (Brunec et al., 2018).

The second study (i.e., the London dataset) included 25 participants (mean age 23.1 years, *SD* = 3.04; 13 female). One additional participant was excluded due to below chance performance on the in‐scan task. High‐resolution T1‐weighted structural scans were acquired using a 1.5 T Siemens Avanto MRI scanner at the Birkbeck‐UCL Centre for Neuroimaging (TR = 12 ms, TE = 5.6 ms, 1 × 1 × 1 mm^3^ resolution). The study was approved by the UCL research ethics committee and the Birkbeck‐UCL Centre for Neuroimaging (BUCNI) ethics committee. Functional data from this experiment have previously been reported (Patai et al., 2017).

In the Toronto study, participants navigated freely by choosing their route between specified start and end points, in contrast to the London study, in which participants made navigational judgments at decision points but could not navigate off‐course (Appendix B). In the Toronto study, participants were required to navigate using arrow keys, such that each keypress advanced their position by one step in the direction of their choice. Therefore, we were able to calculate navigational efficiency, defined as the difference in Euclidean distance to goal with each step. We found a significant relationship between mapping scores and navigational efficiency (*r* = .486, *p* = .004; Figure 1), supporting the notion that higher mapping scores relate to more efficient navigation.

**Figure 1:**
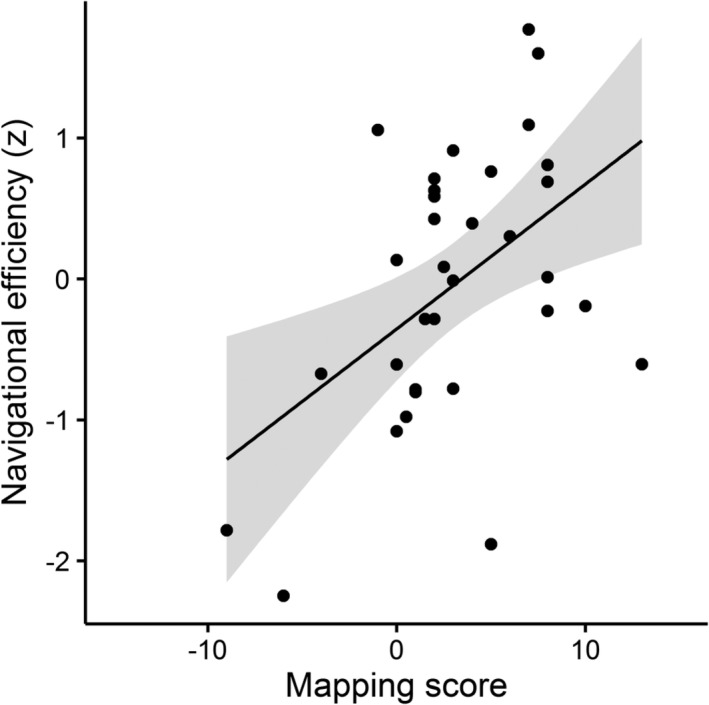
Navigational efficiency significantly correlated with mapping scores in the Toronto dataset. Mapping scores above 0 indicate a preference for map‐based navigation, and scores below 0 indicate a preference for scene‐based navigation. Navigational efficiency was calculated as the change in distance to goal with each step (key press). This measure was converted to *z*‐scores to enable us to combine data across two separate experiments. The shaded area represents 95% confidence intervals around the fitted linear trendline

Participants' hippocampi were extracted using FSL FIRST (Patenaude, Smith, Kennedy, & Jenkinson, 2011), after which they were manually segmented into anterior and posterior portions based on the location of the uncal apex (Poppenk & Moscovitch, 2011). Further following the method presented by Poppenk and Moscovitch (2011), hippocampal volume ratios were calculated by dividing posterior segment volumes by anterior segment volumes. Ratios above 1, therefore, indicate greater pHPC, relative to aHPC, and ratios below 1 indicate greater aHPC, relative to pHPC. There was no significant difference in volume ratios across the two datasets in either the left hemisphere (*t*(56) = −.075, *p* = .940) or the right hemisphere (*t*(56) = .369, *p* = .713). The mean volume ratio in the left hemisphere across both datasets was .984 (*SD* = 0.171), and the mean volume ratio in the right hemisphere was .930 (*SD* = 0.155). The mapping scores in the London study (*M*_NSQ_ = 5.52, *SD*_NSQ_ = 3.66) were significantly higher than those in the Toronto study (*M*_NSQ_ = 3.09, *SD*_NSQ_ = 4.53); *t*(56) = 2.19, *p* = 0.033. The left and right hippocampal volume ratios were then correlated to participants' mapping scores measured by the NSQ across both datasets (*M*_NSQ_ = 4.14, *SD*_NSQ_ = 4.32). In the combined dataset across both studies, there was a significant relationship between right hippocampal volume ratio and mapping (*r* = .397, *p* = .002; Figure 2c), but not between left hippocampal volume ratio and mapping (*r* = .180, *p* = .176; Figure 2b). For illustrative purposes, we also calculated the mean volume ratio (left and right hemispheres combined) and correlated it to mapping scores (Figure 2a). These results suggest higher pHPC/aHPC volume ratios relate to higher mapping scores, providing evidence that posterior hippocampal function relates to navigational strategy.

**Figure 2:**
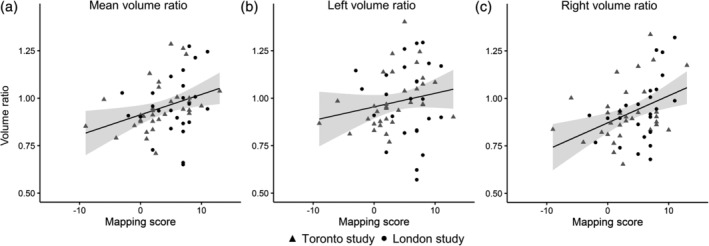
Correlations between (a) mean (left/right average) pHPC/aHPC, (b) left pHPC/aHPC, and (c) right pHPC/aHPC volume ratios and mapping scores. A volume ratio above 1 indicates a larger pHPC, relative to aHPC, and a volume ratio below 1 indicates a larger aHPC, relative to pHPC. The trendlines are plotted for the combined sample (both London and Toronto studies), but individual participants are represented by shapes corresponding to each of the two studies. The shaded areas represent 95% confidence intervals around the fitted linear trendlines

The same pattern of results broadly held when the data were split by study. In the Toronto dataset, there was a significant relationship between both right volume ratio and mapping (*r* = .352, *p* = .044), and left volume ratio and mapping (*r* = .410, *p* = .018). In the London dataset, there was a significant relationship between right volume ratio and mapping (*r* = .482, *p* = .015), but not between left volume ratio and mapping (*r* = −.049, *p* = .815; Figure 2). The relationships observed in the combined sample, therefore, broadly hold up in each of the individual samples with minor variations, though it is important to note that the sample sizes in each of the individual studies may be too small to draw strong conclusions about the relative differences between them.

To control for possible confounds, we ran a series of control analyses. We found no difference between male and female participants in mapping scores (*t*(56) = −1.366, *p* = .177) or volume ratios (right hippocampus: *t*(56) = 0.765, *p* = .447; left hippocampus: *t*(56) = 0.962, *p* = .340). There was no relationship between hippocampal volume ratio and Santa Barbara Sense of Direction Scale (Hegarty, Richardson, Montello, Lovelace, & Subbiah, 2002), designed to measure spatial ability (right hippocampus: *r* = −.097, *p* = .471; left hippocampus: *r* = −.034, *p* = .801), suggesting that our results are not related to general navigational ability but to map use specifically. In the Toronto dataset, we also found no significant relationship between hippocampal volume ratio and navigation efficiency (right hippocampus: *r* = .188, *p* = .294, left hippocampus: *r* = .251, *p* = .160), again supporting the specificity of the link between pHPC/aHPC volume ratios and navigational strategy, but not ability. Last, to control for whole brain volume, we calculated a partial correlation predicting mapping scores from pHPC/aHPC volume ratio while controlling for whole‐brain cerebrospinal fluid, white matter, and grey matter volume estimates. The partial correlation was significant for the right volume ratio (*r* = .447, *p* < .001), but not left volume ratio (*r* = .177, *p* = .196).

Together, these analyses suggest that pHPC/aHPC volume ratios, particularly in the right hemisphere, are related to greater reliance on cognitive maps. This relationship holds up even after controlling for grey matter, white matter, and CSF volumes, and appears to be specific to navigational strategy, but does not extend to in‐task navigational ability. Individuals with larger posterior, relative to anterior, hippocampal volumes in the right hemisphere tended to rate their use of map‐based navigational strategies more highly. Map‐based spatial navigation requires an integrated, fine‐grained spatial representation (Weisberg & Newcombe, 2018) and the use of flexible behavioral strategies when planning novel goal‐directed routes (Wolbers & Hegarty, 2010). Constructing a novel route within a learned environment shares similarities with episodic reconstruction, in that both involve the reinstatement of a broad episodic context and retrieval of individual details (Brunec, Moscovitch, & Barense, 2018). In line with existing evidence that recollective ability relates to larger hippocampal volume ratios (Poppenk & Moscovitch, 2011), the present results indicate convergence across mnemonic and spatial domains. This interpretation is consistent with recent theoretical views proposing that the pHPC supports fine‐grained representations while the aHPC supports more coarse‐grained representations (Brunec et al., 2018; Howard et al., 2014; Milivojevic & Doeller, 2013; Poppenk et al., 2013; Robin & Moscovitch, 2017; Sheldon & Levine, 2016). In recent work from our teams, we find functional neuroimaging evidence for the distinction between map‐based and scene‐based navigation, such that higher mapping scores relate to more variable voxelwise dynamics in pHPC (Brunec et al., 2018), and more pronounced goal‐distance‐coding responses (Patai et al., 2017).

While our effects replicate across two independent samples, the magnitude of the correlations in both studies was moderate. This finding likely signifies that other factors mediate the relationship between hippocampal volume ratios and self‐reported navigational strategies. These other factors might include variations in the ability of participants to reflect accurately on their navigational styles and variance in navigational tendencies depending on the experience and familiarity with an environment. The cities where the two samples of participants resided also have very different configurations: Toronto has a highly regular grid‐like structure and London does not. This difference in the environments experienced by participants over their lifetimes may also relate to a difference in navigational styles (Spiers & Maguire, 2007) and, therefore, the difference in mean mapping scores across the two samples. Future work is needed to relate individual differences in navigational abilities to differences in environmental configurations, especially since differences have been observed between different measures of space syntax and aHPC and pHPC activity (Javadi et al., 2017). While we cannot infer causation based on these correlational data, evidence that pHPC/aHPC ratios increase with experience in London taxi drivers implies that as these specialized populations develop extremely proficient mapping abilities, their hippocampal volumes may change accordingly, although evidence suggests that change in pHPC may occur on a more rapid timescale than in aHPC (Maguire et al., 2000, 2006; Woollett & Maguire, 2011; Woollett, Spiers, & Maguire, 2009). Whether more extensive training would lead to a trade‐off between pHPC and aHPC volumes, and whether a similar mechanism might operate in the general population should be explored in future longitudinal studies of mapping abilities and changes in aHPC and pHPC volumes. Existing evidence suggests that recently, but not remotely, learned environments and routes necessarily require or activate the hippocampus (Hirshhorn, Grady, Rosenbaum, Winocur, & Moscovitch, 2012; Moscovitch et al., 2005; Patai et al., 2017; Rosenbaum et al., 2000; Rosenbaum, Ziegler, Winocur, Grady, & Moscovitch, 2004). Although the present data suggest that a larger pHPC/aHPC ratio is associated with implementing a map‐based strategy, it is not clear whether it is a necessary condition for using cognitive maps effectively in remotely learned environments.

Some evidence suggests that right hippocampal volume is predictive of navigational abilities (Nedelska et al., 2012; Schinazi et al., 2013), though a study has also reported a significant relationship between right aHPC volume and topographical memory (Hartley & Harlow, 2012). In a subset of the data reported here, we found that navigational efficiency was related to self‐reported use of cognitive maps, but not directly to hippocampal volume ratios. This observation is consistent with prior evidence showing no link between navigational abilities and hippocampal volume in the general population (Maguire et al., 2003; Weisberg, Newcombe, & Chatterjee, 2018). The latter finding raises the possibility that increased pHPC volumes in taxi drivers reflect their spatial navigation strategy rather than ability alone. The inconsistencies in these results may stem from the differences in the metrics of navigational abilities being studied, which warrants further investigation.

These results could have implications for understanding AD and mild cognitive impairment (MCI). As spatial disorientation is an early and common symptom of AD, the relationship between navigational strategy and the detection of pathological aging patterns needs to be explored in future work (Coughlan, Laczó, Hort, Minihane, & Hornberger, 2018). Recent research has found that atrophy of the pHPC in cases of MCI and AD is associated with tau‐pathology, Aβ‐pathology and declines in verbal and spatial memory (Lindberg et al., 2017; Llado et al., 2018), whereas nonpathological aging has generally been associated with mid‐ or anterior, but not posterior, volume reductions (Malykhin, Huang, Hrybouski, & Olsen, 2017; Rajah, Kromas, Han, & Pruessner, 2010; Ta et al., 2012). Thus, changes to pHPC/aHPC volume ratios could potentially serve as indicators of MCI or AD vulnerability, and accompany changes in spatial memory and navigation strategy.

## APPENDIX A NAVIGATIONAL STRATEGIES QUESTIONNAIRE

The navigational strategies questionnaire, used to assess propensity for map‐based navigation, is reproduced here:

Note: Each response had an answer corresponding to a map‐based navigation strategy or characteristic (indicated in bold) and one corresponding to a non‐map/scene‐based strategy (underlined). The mapping tendency was calculated as the difference between the number of map‐based answers and non‐map‐based answers. Some questions had a third alternative, which was not coded.

This questionnaire contains questions about your experience navigating, the strategies you use, and what helps you to navigate. Circle the answer for each question that best describes how you navigate, or describe your answer in the space beside “Other” if neither applies.

1. When planning a route, do you picture a map of your route or do you picture scenes of what you will see along the way?
  **Map** Scenes Other: __________________
2. Do you consider yourself a good navigator?
  **Yes** No
3. Do you find it easy to read and use maps?
  **Yes** Somewhat No
4. How often do you get disoriented while finding your way around?
  Very often Somewhat often **Very rarely**
5. When thinking about a familiar street, how well can you picture the buildings along it?
  Very clearly Somewhat clearly **Hardly at all**
6. Would you give directions to a friend in terms of landmarks (i.e., when you see the subway stop, turn left?) or in terms of map directions (i.e., walk north four blocks, then turn left?)?
  Landmarks **Map Directions** Other: ___________________
7. Do you picture travelling a route on street level or from a bird's eye view?
  Street‐level **Bird's Eye View** Other: __________________
8. When navigating in an area you know well, do you usually just know where to go or do you need to look around at the surroundings to decide (e.g., coming out of a subway station)?
  **Know it** Some of each Need to look around
9. When travelling along a new route, do you usually remember what buildings you've passed?
  Yes Somewhat **Rarely**
10. Would you prefer to navigate using a list of directions or a map?
  Directions **Map** No preference
11. Do you use landmarks (i.e., familiar buildings) to orient yourself when navigating?
  Often Sometimes **Rarely**
12. Do you find you're flexible navigating along routes (i.e., you can take new shortcuts easily), or do you prefer to follow the same path every time?
  **Flexible** Somewhat flexible Prefer the same route
13. How easily could you draw a map of an area of the city that you know well?
  **Very easily** Somewhat easily Not easily
14. Do you think that you navigate by following a mental map, or working on scene at a time?
  **Maps** Scene at a time Other

## APPENDIX B NAVIGATION TASK DESCRIPTIONS

The Toronto study included data from two experiments in which participants navigated along routes in a virtualized version of Toronto using images from Google Street View. The functional data from one of the experiments were previously reported, along with more detail about the task (Brunec et al., 2018). In this experiment, participants actively navigated along 12 long routes (2–10 km) with different degrees of familiarity and 3–4 turns. The participants constructed the routes with the experimenter ahead of the experiment. They were allowed to create routes anywhere within a 42 × 27 km region of Toronto. In the previously unpublished Toronto experiment, participants navigated in a much smaller area, constrained to the downtown University of Toronto campus (430 × 340 m). In this experiment, participants were required to complete a large number of short routes across campus, each containing at least two turns. In both experiments, because participants were navigating actively, we were able to calculate the decrease in Euclidean distance to goal with each step (each key press the participants made to move in the environment) as a measure of navigational efficiency. However, because of the structure of the routes and because the navigated area was rectangular in the latter experiment, the decrease in Euclidean distance to goal per step was higher than in the first experiment. To be able to combine data across the two experiments, we *z*‐scored the values within each experiment and correlated the resulting *z*‐scores to mapping scores.

The functional data from the London study were previously reported, along with more detail about the task (Patai et al., 2017). In this study, participants navigated along routes constructed from Google Street View images, using the same software as in the Toronto study. Participants completed 8 familiar and 8 unfamiliar routes. Navigation in this task was not active – instead, participants were passively guided between decision points and were required to make direction judgments when they reached junction points or when new goals were presented. As the participants did not navigate actively and their responses did not affect the actual trajectory of the path, the calculation of a similar navigational efficiency measure as in the Toronto study was not possible.
